# Application of the National Comprehensive Cancer Network-distress thermometer in pediatric patients during autologous and allogeneic hematopoietic stem cell transplantation and relationship to blood parameters of the stress axis

**DOI:** 10.1007/s00432-023-05300-1

**Published:** 2023-09-07

**Authors:** Carmen Isolde Malaval, Karin Melanie Cabanillas Stanchi, Dustin Werle, Stefanie Thiel, Melanie Gansel, Peter Lang, Rupert Handgretinger, Jennifer Svaldi, Michaela Döring

**Affiliations:** 1 Department I-General Pediatrics, Hematology and Oncology, University Hospital Tübingen-Children’s Hospital Tuebingen, Hoppe-Seyler-Str. 1, 72076 Tübingen, Germany; 2 Clinical Psychology and Psychotherapy, University of Tübingen, Schleichstr. 4, 72076 Tübingen, Germany

**Keywords:** Distress thermometer, Pediatric patients, Stress hormones, Hematopoietic stem cell transplantation, Cortisol, Psychological burden

## Abstract

**Purpose:**

Hematopoietic stem cell transplantations (HSCT) are extremely stressful procedures for pediatric patients. The activation of the hypothalamic pituitary adrenocortical axis (HPA) can influence the immune system negatively and therefore the overall outcome. The distress thermometer (DT) is an easy to use tool for the self-assessment of perceived distress.

**Methods:**

In this prospective study, a DT with an attached problem list was used in 40 pediatric patients undergoing HSCT and in one parent of each patient. The patients were aged 10–18 years. The patients' cortisol, thyroid stimulating hormone, free triiodothyronine and thyroxine levels were measured regularly during the in-patient stay.

**Results:**

After admission to the hospital, the stress levels of the pediatric patients and their parents increased and reached their maximum on the day of HSCT. The overall stress values of the parents were higher than those of their children. There was a significant difference in the parents’ stress levels on the day of HSCT, as compared to their stress levels on other days. The mean cortisol values of the pediatric patients also increased after admission, reaching significant elevated levels above the upper normal limit 1 week after HSCT and on discharge day. Although the pediatric patients experienced mainly exhaustion, especially on the day of transplantation, their parents mainly felt worry and anxiety. Interestingly, the rate of worry among children increased in the post-transplant period and reached its maximum on the day of discharge.

**Conclusions:**

In summary, a significantly increased stress level is shown for both the patients and their parents. This is reflected for the patients both in the DT scores and in the increased cortisol values. For the parents, the focus is primarily on worry and anxiety, for the patients primarily on exhaustion and worry.

## Background

Hematopoietic stem cell transplantation (HSCT) is a curative therapy for various malignant and nonmalignant diseases (Gratwohl et al. 2010). The psychological impact on children receiving HSCT is similar to that of children receiving chemotherapy (Lazor et al. 2017, 2020). In both cases, children have to cope for a long period of time with the fact that they are not in their familiar home environment, they cannot maintain the same social contacts as previously, and they also have to endure the side effects associated with the treatment. Especially in the case of malignant primary diseases, fears of death, or relapse cause further psychological stress (Lazor et al. 2017; Meyers et al. 1994). This psychological stress can lead to anxiety and depression, which can worsen both the quality of life and survival rate of the patients (Lauer 2015; Wiener et al. 2017). Psychoneuroimmunological studies provide evidence that depression and anxiety can negatively affect the function of the immune system (McGregor et al. 2013). Thus, psychological stress during HSCT could directly influence the outcome by delaying the regeneration of the immune system. There is evidence that experiencing severe anxiety and depression a short time after initial diagnosis can lead to persistence of these symptoms (Myers et al. 2014). In addition, the psychological burden during HSCT can cause negative long-term effects especially in adolescent patients, and can lead to health impairment and unhealthy behavior even years after the disease has been overcome (Gianinazzi et al. 2013; Lauer 2015). Intensified psycho-oncological care is therefore strongly indicated immediately during or after the occurrence of such high psychological stress.

Because uniform guidelines for early assessment of stress in hemato-oncology pediatrics are still missing, anxiety and depression are often diagnosed and treated too late in pediatric patients (Lazor et al. 2020). For this reason, a screening test for mental distress that can be easily implemented in everyday clinical practice should be established in the field of pediatric oncology (Lazor et al. 2020; Seelisch et al. 2018). The United States National Comprehensive Cancer Network (NCCN) distress thermometer (DT) was developed according to these needs and it allows for a quick and easy survey of distress (Jacobsen et al. 2005). Previous studies have shown that its use is reliable, valid and acceptable in pediatric cancer patients (Patel et al. 2010, 2011, 2014, 2021; Schulte et al. 2019; Wiener et al. 2017). With the help of the DT, the child can classify his or her subjectively experienced stress on a visual analog scale with the subdivisions 0 (no stress) to 10 (extreme stress). In both adult and pediatric oncology patients, this test has been used successfully in the daily routine to identify those patients who should be evaluated in more detail (Kazak et al. 2015; Patel et al. 2010; Wiener et al. 2017). A German version has been validated for adult patients (Mehnert et al. 2006).

In this prospective single center study, the DT was used to assess distress before, during, and after allogeneic HSCT in 40 pediatric patients with malignant and nonmalignant diseases, aged 10–18 years. The aim of the study was to assess the psychological distress of both pediatric patients and their parents during the course of HSCT up to discharge. In addition, we investigated whether the psychological stress follows a certain temporal pattern in the course of HSCT and what subjective reasons for an increased stress load were given at certain points in time. Hereby, it was investigated whether it is possible to determine potential critical time points and external factors in the course of HSCT. This could further increase the rate of early diagnosis of mental distress in children and early initiation of appropriate psychotherapeutic support.

## Methods

### Study conception and design

In this prospective monocentric study, 40 pediatric HSCT patients between 10 and 18 years of age, and one parent of each patient, were prospectively and consecutively enrolled at in-patient admission for scheduled HSCT between January 2019 and December 2020.

Patients were excluded if they were under 10 years of age, or if the patients and/or their parents did not have sufficient German or English language skills to complete the questionnaires used in this study. Furthermore, the patients were excluded from the study if they had already been diagnosed with a mental illness prior to HSCT.

The observation period was defined as the period from one day before start of the conditioning regimen until the date of discharge after HSCT.

### The distress thermometer

The NCCN-DT is a screening tool used to assess distress in patients with hemato-oncological malignancies, developed by the United States National Comprehensive Cancer Network (NCCN). It is a single item screening instrument with an eleven-point visual analogue scale on what the subjectively experienced distress level can be rated from 0 (no distress) to 10 (extreme distress). A cutoff score ≥ 5 is internationally recommended as an indicator for a distressed patient. In addition, a problem list identifies potential sources of distress. We have made slight changes to make it easier for children and young people to see the categories: the thermometer has been colored from green (no stress) to red (maximum stress). The questions for the problem scales were not changed.

The children and one parent (always the same parent; the parent who was admitted to the in-patient stay) per child were given a distress diary with the DT included on every page to self-assess their distress level. The problem lists were handed out at the following time points to analyze the possible cause of distress: day of admission, five days before HSCT (day − 5), the day of HSCT (day 0), day + 7, day + 14, day + 21, day + 28, day + 35, day + 42, day + 50, day + 60 after HSCT and the day of in-patient discharge. Using these stress diaries with the DT and the problem lists, the reported stress levels were recorded and evaluated during the time period on the ward on the day of in-patient admission, day − 5, day 0, day + 7, day + 14, day + 21, day + 28, day + 35, day + 42, day + 50, day + 60 after HSCT and the day of in-patient discharge.

### Determination of blood parameters

The stress parameters cortisol, the thyroid stimulating hormone (TSH), free triiodothyronine (fT3) and thyroxine (fT4) were determined in the blood of the pediatric patients at the same time points for analysis of the DTs to compare the results. TSH, as well as fT3 and fT4 were quantified in heparinized peripheral blood using enzyme immunoassays, i.e. the ADVIA Centaur^®^ XPT Immunoassay-System (Siemens-Healthcare GmbH, Erlangen, Germany).

Cortisol is a hormone of the stress response and recruits energetic compounds and molecules through activating catabolic processes. Cortisol was determined in the blood plasma using the corticosterone (Rat/Mouse 9 ELISA Kit from DRG Instruments GmbH, Marburg, Germany). The sampling and preparation of the peripheral blood taken from routine blood sampling were performed according to the manufacturer information, including the determination of negative and positive control samples.

All blood parameters were determined from blood samples taken as part of routine diagnostics, which took place daily in the morning between 6 and 7 am. An influence of the circadian rhythm on these parameters could thus be minimized. No cortisol parameters were recorded during steroid substitution. Results were determined in the blood of the pediatric patients at the same time points for analysis of the DTs in order to compare the results.

### Statistical methods

All data were tested for normal distribution using the Shapiro–Wilk test. Because of significant deviations from normality, data of the DT were then tested with the Kruskal–Wallis test and, if significant, the *p* value was then finally determined using the Dunn’s multiple comparison test. The DT data were reported as median ± 25th/75th percentile of the study cohort on the respective observation days. A number in the according symbol in the graphs represent the sample size of the analyzed data set.

Laboratory data were analyzed descriptively (mean, median, range, 25th percentile, 75th percentile, minimum, and maximum) and tested for normal distribution using the Shapiro–Wilk normality test. Multiple comparisons between baseline values and values at different observation days were analyzed with a 2-way ANOVA analysis of variance. Significant deviations above or below the upper or lower normal limits were detected using the Wilcoxon-signed rank test. The results are displayed as mean ± standard error of the means (SEM) in the graphs.

The maximum problem awareness of mental and somatic problem percentages was displayed as mean ± 95% CI. Comparisons between results of parents and patients were performed using an unweighted Cohen’s Kappa. The agreement measures were defined as < 0.00 (poor), 0.00–0.20 (slight), 0.21–0.40 (fair), 0.41–0.60 (moderate), 0.61–0.80 (substantial), 0.81–1.00 (almost perfect).

All *p* values were adjusted for multiple testing. *p* values of < 0.05 (*), *p* < 0.01 (**), *p* < 0.001 (***), and *p* < 0.0001 (****) were defined as statistically significant.

The collection of laboratory data was performed using the laboratory information system Swisslab LAURIS (version 16.10.05.5, Roche Diagnostics IT Solutions GmbH, Berlin, Germany). All medical reports derived from the SAP system of the University Children’s Hospital Tübingen (SAP Netweaver SAP GUI for Windows, Version 7300.3.10.1084, SAP SE, Walldorf, Germany). The data were collected using Microsoft Excel (Microsoft Excel 2010 Version 14.0 for Windows, Microsoft Germany GmbH, Munich, Germany). Graphs and statistical tests were created with GraphPad Prism for Windows, version 9.4, 2022 (GraphPad Software Inc., La Jolla, CA, USA).

## Results

### Patients characteristics:

Patients characteristics are summarized in Table 1. The patient group consisted of 40 pediatric patients who received an HSCT because of a hematologic–oncologic malignant or nonmalignant underlying disease. From these patients, 22 patients (55.0%) were male, 18 patients (45.0%) were female. The median age was 14.6 years (range 10–18 years). Patients received grafts from matched sibling donors (MSD; *n* = 13; 32.5%), matched unrelated donors (MUD; *n* = 15; 37.5%), mismatched family donors (MMFD; *n* = 7; 17.5%), and themselves (autologous; *n* = 5; 12.5%). The conditioning regimen was in 37 out of 40 patients myeloablative (92.5%) and in three cases intensity-reduced (7.5%), composite in one case of cyclophosphamide (4 × 50 mg per kilogram (mg/kg)) and antithymocyte globulin (ATG; 3 × 10 mg/kg), in one case of fludarabine (3 × 40 mg per square meter (mg/m^2^)), thiotepa (15 mg per kilogram (mg/kg)), total lymphoid irradiation (TLI, 1 × 7 Gray (Gy)) and ATG (2 × 15 mg/kg) and in the third case of fludarabine (5 × 30 mg/m^2^), total body irradiation (TBI; 4 Gy), Thiotepa (2 × 5 mg/kg), ATG (6 mg/kg) and post-transplant cyclophosphamide (2 × 50 mg/kg). Out of the 40 patients, 17 (42.5%) suffered from acute leukemia or myelodysplastic syndromes and four patients (10.0%) had a lymphoma. Furthermore, seven patients (17.5%) were diagnosed with a nonhematologic solid tumor and twelve patients (30.0%) had a nonmalignant underlying disease. During the clinical course 17 patients (42.5%) suffered from sepsis or systemic inflammatory response syndrome (SIRS) and viremia. An acute graft-versus-host disease (aGvHD) grade 1 or 2 occurred in eleven patients (27.5%). There was no case of higher grade aGvHD during the observation period. A veno-occlusive disease (VOD) was diagnosed in seven patients (17.5%) within the clinical course and three patients (7.5%) had a graft failure. The median observation period was 51.5 days (range 24–155 days).

**Table 1:** Patient characteristics

Patient characteristic	Number (%)
**Sex**	
Male	22 (55.0)
Female	18 (45.0)
**Age [years]**	
Median (range)	14.6 (10.0–18.0)
**HSCT donor**	
Matched sibling donor	13 (32.5)
Matched unrelated donor	15 (37.5)
Mismatched family donor	7 (17.5)
Autologous	5 (12.5)
**Diagnosis**	
*Malignant diseases*	
Acute lymphoblastic leukemia and relapse	9 (22.5)
Acute myeloid leukemia and relapse	5 (12.5)
Ewing’s sarcoma	3 (7.5)
Myelodysplastic syndromes	3 (7.5)
Morbus Hodgkin	2 (5.0)
Neuroblastoma	2 (5.0)
Osteosarcoma	1 (2.5)
Nephroblastoma	1 (2.5)
Anaplastic large cell lymphoma	1 (2.5)
Peripheral T cell lymphoma	1 (2.5)
*Nonmalignant diseases*	
Sickle cell anemia	7 (17.5)
Thalassemia	2 (5.0)
Severe aplastic anemia	2 (5.0)
Severe combined immunodeficiency	1 (2.5)
**Conditioning regime**	
Reduced-intensity conditioning	3 (7.5)
Myeloablative conditioning	37 (92.5)
**Complications after HSCT**	
Sepsis/SIRS	17 (42.5)
aGvHD grade 1 or 2	11 (27.5)
aGvHD grade 3 or 4	0 (0.0)
VOD	7 (17.5)
Viremia	17 (42.5)
Graft failure	3 (7.5)

*aGvHD* acute graft-versus-host disease, *HSCT* hematopoietic stem cell transplantation, *n* sample size, *SIRS* systemic inflammatory response syndrome, *VOD* veno-occlusive disease

### Analysis of the distress thermometer

**Fig. 1:**
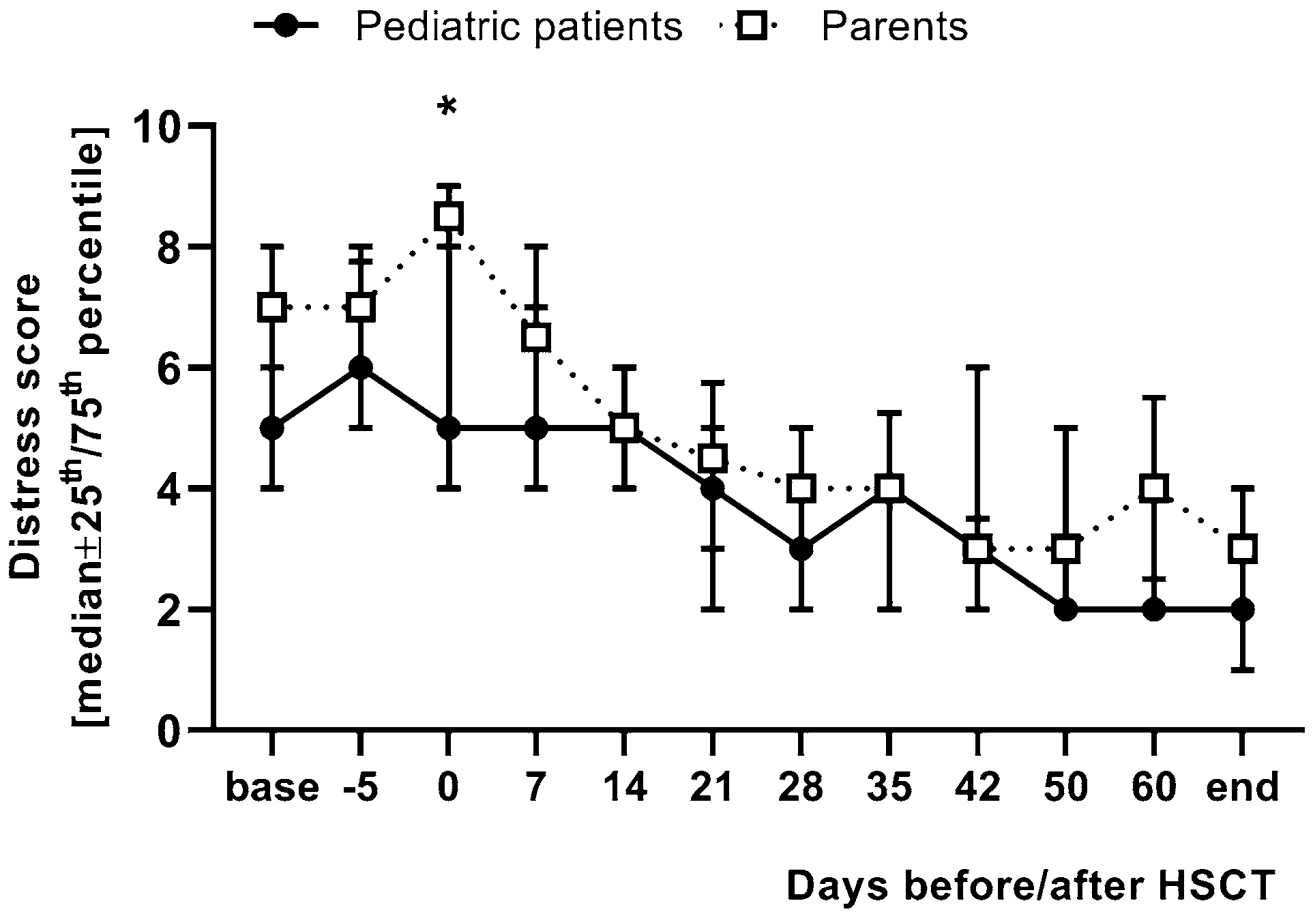
Distress thermometer. The figure shows the NCCN distress thermometer scores (median ± 25th/75th percentile) of 40 pediatric patients undergoing hematopoietic stem cell transplantation (HSCT) with one parent each collected, on the respective observation days before, during and after HSCT. Base = day of admission, end = day of discharge. DT of the pediatric patients: the median values of the DT of the pediatric patients at the time points of 4 weeks, day + 50 after HSCT and on the day of discharge were significantly below the median baseline value on the day of admission (day + 28 vs. base: *p* = 0.0224; day + 50 vs. base: *p* = 0.0057; day of discharge vs. base: *p* < 0.0001*).* The median values of the DT of the parents from the time point 3 weeks after HSCT until the day of discharge were significantly below the median DT baseline value (day + 21 vs. base: *p* = 0.0016; day of discharge vs. base: *p* < 0.0001). The DT values of the parents were significantly above the DT values of the pediatric patients on the day of HSCT (day 0; *p* = 0.0157)

The median baseline DT value of the patients was 5 (range 3–9). On day − 5 before HSCT, the patients reported a peak median DT value of 6 (range 2–9, DT day − 5 vs. base: *p* = 0.76), followed by a downward trend with a median of five 1 week after HSCT (range 0–9, DT day + 7 vs. base: *p* > 0.99), 5 on day + 14 (range 0–10, DT day + 14 vs. base: *p* > 0.99), 4 on day + 21 (range 0–7, DT day + 21 vs. base: *p* = 0.17), 3 on day + 28 (range 0–8, DT day + 28 vs. base: *p* = 0.0224), 4 on day + 35 (range 1–9, DT day + 35 vs. base: *p* = 0.22), 3 on day + 42 (range 1–10, *p* = 0.0142), 2 on day + 50 (range 2–6, day + 50 vs. base: *p* = 0.0057), 2 on day + 60 (range 1—5, day + 60 vs. base: *p* = 0.0056) and 2 on the day of discharge (range 0—4, DT day of discharge vs. base: *p* < 0.0001). The median values of the DT at the time points of 4 weeks, 6 weeks, day + 50 and day + 60 after HSCT and on the day of discharge were significantly below the baseline value on the day of in-patient admission (Fig. 1).

The median baseline DT value of the parent group was 7 (range 2–9). As in the pediatric patients, the parents showed an increase of DT scores from the day of admission to the day of HSCT, with a peak on the day of HSCT (median 8.5, range 2–10), followed by a decreasing trend in the DT value over the further course of time to day + 7 (median 6.5, range 1–9, DT day + 7 vs. base: *p* > 0.99), day + 42 (median 3, range 1–10, DT day + 42 vs. base: *p* = 0.0015), day + 60 (median 4, range 2–6, DT day + 60 vs. base: *p* = 0.0363) and on the day of discharge (median 3, range 0–4, DT day of discharge vs. base: *p* < 0.0001). The median value of the DT on the day of HSCT was significantly compared to baseline (Fig. 1).

Comparing the two groups, the parents of the patients reported higher DT values than the children themselves at all time points. On the day of HSCT, parents scored significantly higher DT values as compared to their children (*p* = 0.0157) (Fig. 1).

### Laboratory analyses

**Fig. 2:**
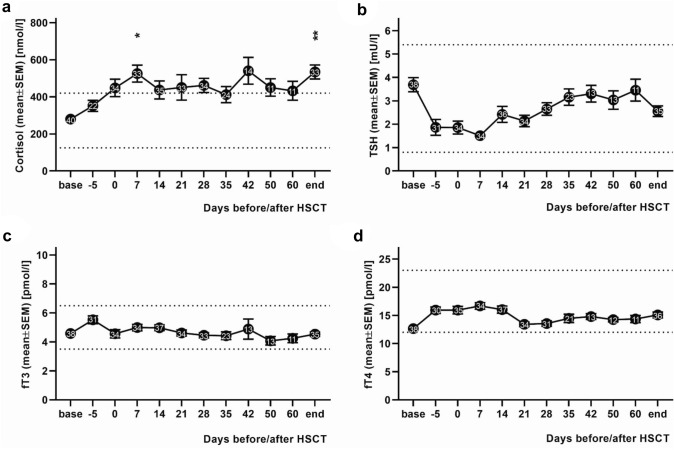
Hormones of the hypothalamic–pituitary axis. The figure shows the laboratory values (mean ± SEM) of the stress hormones cortisol, thyroid stimulating hormone (TSH), free triiodothyronine (fT3) and free thyroxine (fT4) of 40 pediatric patients undergoing hematopoietic stem cell transplantation (HSCT) collected on the respective observation days before, during and after HSCT. Numbers in the plotted data sets indicate sample size. Base = day of admission, end = day of discharge. Note that most patients were discharged between day + 35 and day + 42 after hematopoietic stem cell transplantation (HSCT) (on day + 42, 48% of pediatric patients had already been discharged and on day + 60, 78% had been discharged). **a** Mean cortisol values of the pediatric patients over time. The mean cortisol value was in the normal range on the day of admission and on day − 5 before HSCT, but exceeded the upper limit value on the day of HSCT and remained above upper limit until discharge (*: mean cortisol value day + 7 (525 ± 46 nmol/l) vs. upper normal value: *p* = 0.0293; **: mean cortisol value discharge day (534 ± 37 nmol/l) vs. upper normal value: *p* = 0.0046). **b** Mean TSH values of the pediatric patients over time. The mean TSH values were never outside the normal range. **c** Mean fT3 values of the pediatric patients over time. The mean fT3 values were never outside the normal range. **d** Mean fT4 values of the pediatric patients over time. The mean fT4 values were never outside the normal range. The dashed lines indicate the upper or lower normal limits of the respective measurement parameter

The mean cortisol level of the patients at baseline was 280 ± 21.2 nmol/l and thus within the normal range (125 to 420 nmol/l). The cortisol levels exceeded the upper limit value on the day of HSCT and remained above the upper limit until discharge with a significant difference from the upper normal value on day + 7 after HSCT and on the discharge day (mean cortisol value day + 7 (525 ± 46 nmol/l) vs. upper normal value (420 nmol/l): *p* = 0.0293; mean cortisol value discharge day (534 ± 37 nmol/l) vs. upper normal value: *p* = 0.0046; Fig. 2a).

The mean TSH level of the pediatric patients at in-patient admission was 3.7 ± 0.3 mU/l. The mean TSH values of the patients were never outside the normal range during the in-patient stay (0.8 to 5.4 mU/l). They dropped after admission and reached their lowest point on day + 7 after HSCT. Subsequently, they showed an increasing trend over time (Fig. 2b).

The mean values of fT3 and fT4 at in-patient admission were 4.6 ± 0.2 pmol/l and 12.7 ± 0.4 pmol/l, respectively. The mean values of fT3 and fT4 were never outside the normal range (normal range fT3: 3.5 to 6.5 pmol/l, normal range fT4: 12 to 23 pmol/l) and also showed no upward or downward trend over time (Fig. 2c, d).

### Analysis of the problem lists

**Fig. 3:**
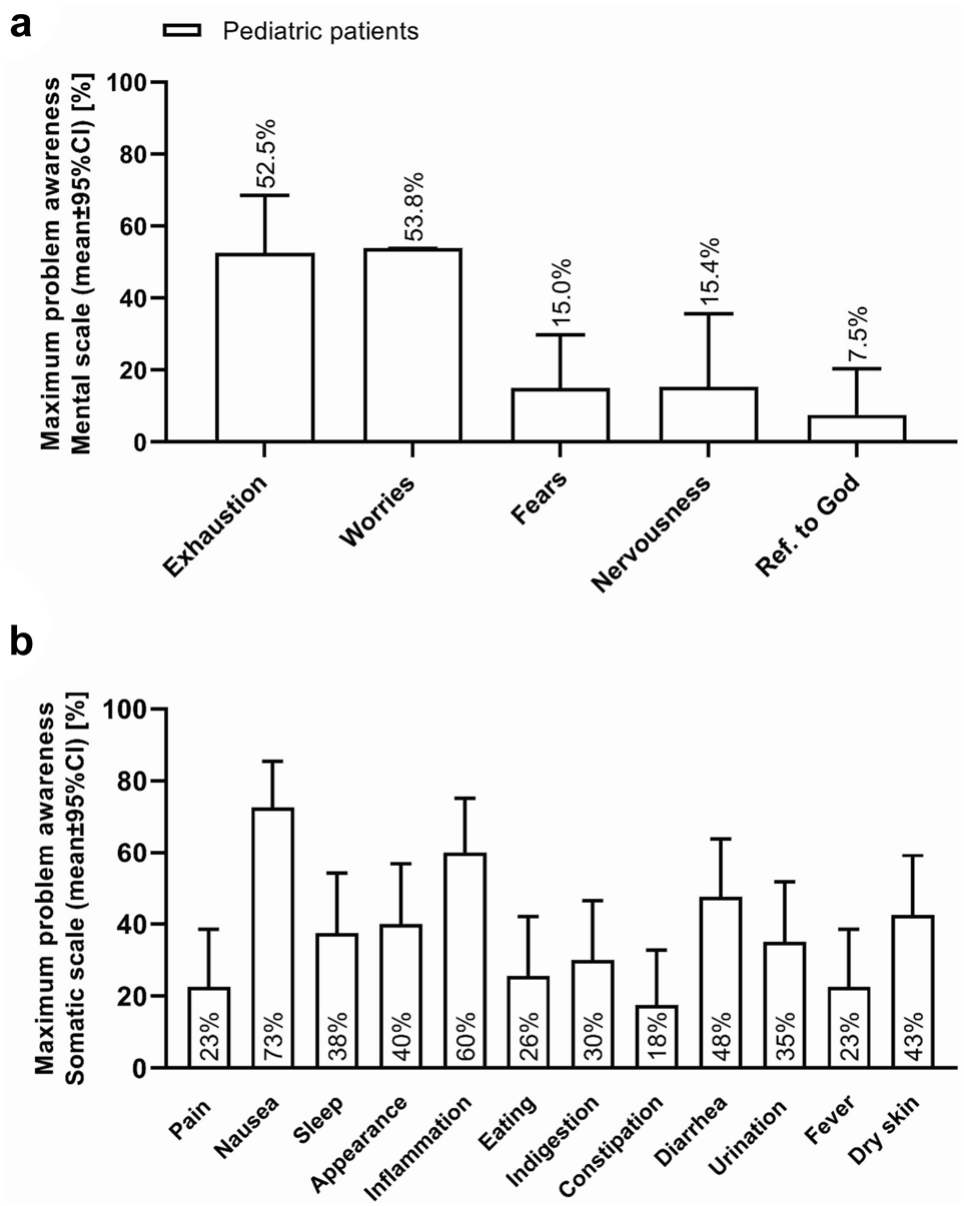
Problem awareness in mental and physical problem regions. The figure illustrates the areas in which the pediatric patients reported problems.** a** The maximum problem awareness (mean ± 95% CI) [%] with regard to the mental problem regions of exhaustion, worries, fears, nervousness, reference to God (ref. to God) of the pediatric patients. In summary, it can be stated that more than half of the children reported problems in the areas of exhaustion and worries. **b** The maximum problem awareness (mean ± 95% CI) [%] with regard to the physical problem regions of pain, nausea, sleep, appearance, inflammation, eating, indigestion, constipation, diarrhea, urination, fever, dry skin of the pediatric patients. In summary, it can be stated that more than half of the children reported problems in the areas of nausea and inflammation

The results of the problem lists are presented in Table 2 and in Fig. 3. In summary, it can be stated that more than half of the children reported problems in the areas of exhaustion, worry, nausea, and inflammation. Among the parents, more than 50% reported problems in the areas of worry and anxiety.

**Table 2:** Analysis of the problem lists

Problem area	Patients		Parents	
	Time point of MPA	MPA [%]	Time point of MPA	MPA [%]
Exhaustion	Day 0	52.5	Day of discharge	10.3
Worry	Discharge	53.8	Day 0	67.5
Anxiety	Day 0	15.0	Day 0	57.5
Nervousness	Discharge	15.4	Day + 14	5.0
Reference to god	Day − 5; day + 14	7.5	Day − 5 to day + 21	5.0
Pain	Day + 7; day + 14	22.5		
Nausea	Day − 5	72.5		
Sleep	Day + 21	37.5		
Appearance	Day − 5	40.0		
Inflammation	Day + 21	60.0		
Eating	Day of discharge	25.6		
Indigestion	Day + 14	30.0		
Constipation	Day + 7	17.5		
Diarrhea	Day 0	47.5		
Changes in urination	Day + 7; day + 14	35.0		
Fever	Day + 14	22.5		
Dry skin	Day + 7	42.5		

Note that day 0 is the day of hematopoietic stem cell transplantation

*MPA* maximum problem awareness

**Fig. 4:**
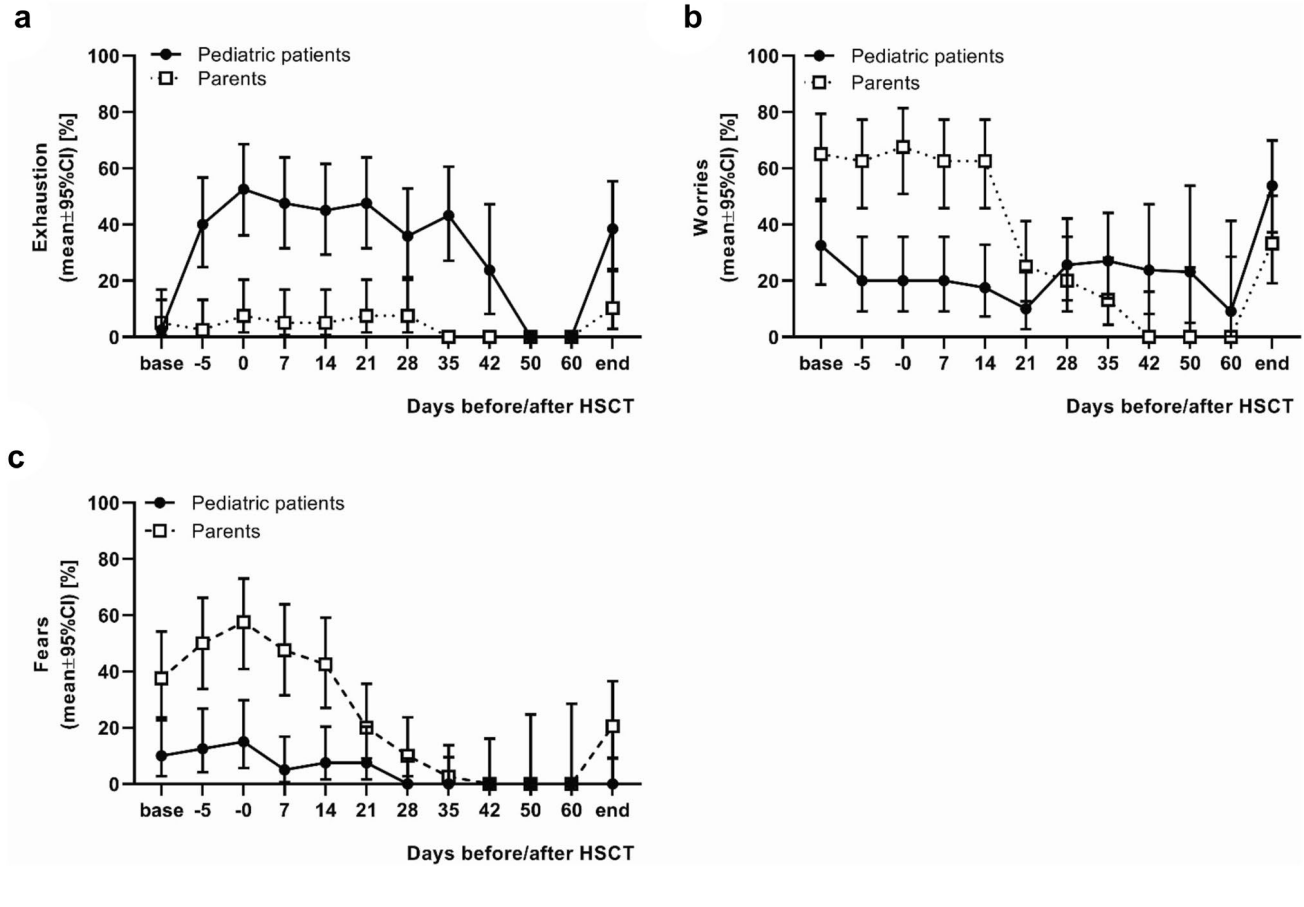
Problem areas exhaustion, worries and fears. The figure shows the percentage of problem awareness in the areas exhaustion, worries and fears of 40 pediatric patients undergoing hematopoietic stem cell transplantation (HSCT) and one parent each collected on the respective observation days before, during and after HSCT. Note that most patients were discharged between day + 35 and day + 42 after HSCT (on day + 42, 48% and on day + 60, 78% of pediatric patients had already been discharged). **a** Problem area exhaustion: More children reported problems in this area than their parents (problem awareness maximum 52.5 vs. 10.3%). This difference reached significance at the time points of day − 5 (*p* < 0.0001), day 0 (*p* < 0.0001), day + 7 (*p* < 0.0001), day + 14 (*p* < 0.0001), day + 21 (*p* < 0.0001), day + 28 (*p* = 0.0021), day + 35 (*p* < 0.0001) and the day of discharge (*p* = 0.0037). The rate of exhaustion in the pediatric patients increased rapidly until the day of HSCT, after which the curve declined very slowly. **b** Problem area worries: the parents initially indicated more problems in this area than the pediatric patients with significant differences at the time points of day of admission (*p* = 0.0036), day − 5 (*p* = 0.0001), day 0 (*p* < 0.0001), day + 7 (*p* = 0.0001) and day + 14 (*p* < 0.0001). However, while the parents' awareness of worries reached its maximum on the day of HSCT (67.5%) and subsequently dropped from day + 21 after HSCT onwards, the rate of worries among pediatric patients increased over time and was significantly higher than that of the parents at day + 42 (*p* = 0.0172). **c** Problem area fears: the parents reported higher problem awareness in this area than the pediatric patients over the entire time course with significant differences at the time points of day of admission (*p* = 0.0039), day − 5 (*p* = 0.0003), day 0 (*p* < 0.0001), day + 7 (*p* < 0.0001), day + 14 (*p* = 0.0003), day + 28 (*p* = 0.0427), and the day of discharge (*p* = 0.0028). In both groups, the maximum percentage of problem perception was the highest on the day of HSCT (children 15.0% vs. parents 57.5%) and subsequently decreased except for the day of discharge, at which time the number of parents perceiving fears increased again

Regarding the problem area of exhaustion over time, it is noticeable that more children reported problems in this area than their parents. This difference reaches significance at the time points of day − 5 (*p* < 0.0001), day 0 (*p* < 0.0001), day + 7 (*p* < 0.0001), day + 14 (*p* < 0.0001), day + 21 (*p* < 0.0001), day + 28 (*p* = 0.0021), day + 35 (*p* < 0.0001), day + 42 (*p* = 0.0172) and the day of discharge (*p* = 0.0037). The rate of exhaustion in the pediatric patients increased rapidly until the day of HSCT, after which the curve tended to decline slowly (Fig. 4a).

Concerning the problem area of worries, it is noticeable that the parents initially indicated more problems in this area than the pediatric patients with significant differences at the time points of day of admission (*p* = 0.0036), day − 5 (*p* = 0.0001), day 0 (*p* < 0.0001), day + 7 (*p* = 0.0001) and day + 14 (*p* < 0.0001). However, while the parents' awareness of worries reaches its maximum on the day of HSCT (67.5%) and subsequently drops from day + 21 after HSCT onwards, the rate of worries among patients increases over time and is significantly higher than that of the parents at day + 42 (*p* = 0.0172) (Fig. 4b).

The awareness of problems in the area of fears was higher among the parents than among the patients over the entire time course with significant differences at the time points of day of admission (*p* = 0.0039), day − 5 (*p* = 0.0003), day 0 (*p* < 0.0001), day + 7 (*p* < 0.0001), day + 14 (*p* = 0.0003), day + 28 (*p* = 0.0427), and the day of discharge (*p* = 0.0028). In both groups, the maximum percentage of problem perception was the highest on the day of HSCT and subsequently decreased except for the day of discharge, at which time the number of parents perceiving fears increased again (Fig. 4c).

## Discussion

In this prospective study, we investigated the psychological distress of pediatric patients undergoing HSCT in our hematological–oncological transplant ward using the DT, a questionnaire regarding problem perception in both physical and psychological areas and also measured the stress hormones cortisol, TSH, fT3, and fT4.

At the beginning after admission to the transplant ward, the patients experienced increasing stress which reached its maximum on the day of HSCT with a median DT value of 5, which decreased over time to a median DT value of 2 on the day of discharge. The stress perception of the parents was the same; their DT values also increased after admission to reach their maximum on the day of the HSCT, thereafter decreasing again until the day of discharge. The median DT values of the parents were higher overall than those of the children, with a significant difference on the day of HSCT (8.5 vs. 5). This result fits with the fact that in previous studies on pediatric patients undergoing HSCT, the patients generally rated their quality of life higher than their parents (Feichtl et al. 2010).

In line with the DT, the stress hormone cortisol also showed an increase from the day of admission. The increase over the upper normal limit reached significant levels on day + 7 and on discharge day. This could indicate that discharge is associated with uncertainty, excitement, and stress. The fact that cortisol levels were above the upper normal limit from the day of HSCT until the day of discharge indicates that the HSCT and the post-transplant period are very stressful and drastic times. Studies have shown that the activation of the hypothalamic pituitary adrenocortical (HPA) axis is associated with reduced DNA repair mechanisms, accelerated tumor growth and angiogenesis via increased release of vascular endothelial growth factor and interleukin-6, invasion of malignant cells by an increased production of matrix metalloproteinases and also delayed wound healing (Antoni et al. 2006; Colon-Echevarria et al. 2019; Costanzo and Lutgendorf 2011; Dai et al. 2020). Studies have also found that stress and depression reduce the activities of cytotoxic T cells and natural killer cells, so that both the development and progression of tumors appear to be favored by stress (Vissoci Reiche et al. 2005). These results indicate that increased stress under HSCT can have a negative impact on post-transplant convalescence and even on the overall prognosis (Aldea et al. 2014; Antoni et al. 2006; Costanzo and Lutgendorf 2011).

The blood levels of the thyroid hormones TSH, fT3 and fT4 were never outside the normal range. However, compared to the basal values on the day of admission, the decreased TSH values on day − 5, day 0 and day + 7 could be an indication that stress correlates laboratory-chemically with an activation of the thyroid hormones (Åsberg et al. 2009; Nadolnik 2011).

Consistent with the cortisol level, the pediatric patients experienced increasing exhaustion with a maximum on the day of HSCT, after which the rate of exhaustion tended to decline very slowly. Parents experienced less exhaustion than pediatric patients throughout the whole period. However, they felt more worry and anxiety compared to their children especially on the day of HSCT. Interestingly, the rate of worries among the pediatric patients increased over the course of time and was higher than that of the parents at the time of discharge. This difference in perception of worry is consistent with the results of a study, in which the worries of pediatric patients who received HSCT and the worries of their parents were analyzed. It became apparent that the parents and their children worried in different degrees and also worried about different topics. When compared with their children, more parents indicated strong worries and expressed worries about the impact of the HSCT on their families and about a bad outcome. In contrast, a larger percentage of children reported neither worries nor concerns about not getting back to normalcy (Levine et al. 2020).

Even though only a small proportion of children reported anxiety, the high rate of anxiety among parents should be taken seriously. It was demonstrated in a prospective study of 113 parents of children with leukemia that parental anxiety experienced during their children's cancer treatment, especially among mothers, is predictive of later post-traumatic stress syndrome (Best et al.^2001^).

It is known from other studies that high stress and anxiety levels are already felt before and increase towards the day of HSCT and then slowly decrease. Social support is therefore important for patients (Meyers et al. 1994). Worry, anxiety as well as reduced communication during the acute phase of HSCT have been identified to have a negative impact on health-related quality of life one year after HSCT (Felder-Puig et al. 2006). It has been shown that the rate of maternal depression correlates with a reduced cognitive and educational outcome of pediatric patients after HSCT (Barrera et al. 2008). A study on the effects of maternal stress following HSCT of their children reports significantly increased stress and anxiety levels in the mothers as compared to the normal values of women of the same age from the general population. They conclude that this may limit their ability to emotionally support their children and therefore early psychological support for these mothers is necessary (Nelson and Belyea 1997). The increased stress levels of the parents do not only lead to parents being less supportive of their children during treatment on the ward. It could be shown that increased stress perception during the children's treatment is associated with post-traumatic stress disorders in the parents after the end of the treatment, so that a stressful family situation also exists afterwards (Best et al. 2001).

In a study on the psychological consequences for parents of children who have received HSCT, a subgroup of parents was found to have increased stress levels even years after HSCT (Roberto Riva 2014).

In adult breast cancer patients, studies have already shown that concomitant, intensified psychological intervention during therapy reduces the risk of recurrence. In patients with gastrointestinal cancer, those who received intensified psychotherapeutic treatment during cancer therapy had an improved long-term survival (Andersen et al. 2008; Giese-Davis et al. 2011; Kuchler et al. 2007).

### Limitations of the study

The stress diaries were given to only one parent—either the mother or the father. It is possible that the perception of the stress level and also the different problem lists differ between the parents. This may result from gender differences or the individual parent’s own coping strategies and levels of resilience. These aspects should be investigated in more detail in future studies.

The in-patient stay during HSCT is quite heterogeneous depending on the course of therapy and occurring complications. Most of the patients are discharged between day + 35 and day + 42 after HSCT, but the range with the discharge day occurring between day + 17 and day + 148 after HSCT is quite wide. Of course, this has an influence on the patients' stress perception, whether they are still in in-patient treatment or already at home. For this reason, data were collected exclusively during the in-patient stay in order to maintain as consistent a setting as possible. As a result, the sample size decreases towards later time points. Differences in stress perception during the in-patient stay or during the time afterwards should be further investigated.

The DT has already been used successfully for a long time in adult oncology to assess stress levels, is therefore very well investigated according to the comprehensive studies, and is widely used due to its quick and uncomplicated application. Few studies have proven its feasibility, validity and specificity in pediatric cancer patients, however not in German and not in the HSCT context (Patel et al. 2010, 2011, 2014). Therefore, our aim was to investigate the implementation and the benefit of the NCCN-distress thermometer for our patients.

## Conclusion

In the present prospective study evaluating stress using the distress thermometer and assessment scales, it was shown that in the pretransplant phase the stress levels of both pediatric patients and their parents increase until the day of HSCT with the parents experiencing significantly more stress than their children on that day. Over the course of the post-transplant period, the rate of worry in the children increases and their cortisol levels also remain in a range above the upper normal limit until discharge. These results suggest an early and continuous intervention, especially up to the day of HSCT both in psycho-oncological counseling with parents and in intervention with the pediatric patients.

## Data Availability

The data that support the findings of this study are available upon reasonable request from the corresponding author. The data are not publicly available due to privacy or ethical restrictions.

## Abbreviations

aGvHD: Acute graft versus host disease
ATG: Antithymocyte globulin
DT: Distress thermometer
fT3: Free triiodothyronine
fT4: Free thyroxine
gy: Gray
HPA: Hypothalamic pituitary adrenocortical axis
HSCT: Hematopoietic stem cell transplantation
mg/kg: Milligram per kilogram
mg/m^2^: Milligram per square meter
MMFD: Mismatched family donor
MSD: Matched sibling donor
MUD: Matched unrelated donor
mU/l: Milli units per liter
NCCN: National Comprehensive Cancer Network
nmol/l: Nanomol per liter
n.s.: Not significant
pmol/l: Picomol per liter
SIRS: Systemic inflammatory response syndrome
TBI: Total body irradiation
TLI: Total lymphoid irradiation
TSH: Thyroid stimulating hormone
VOD: Veno-occlusive disease

## References

[CR1] Aldea MCL, Tomuleasa C, Crivii C (2014). The role of depression and neuroimmune axis in the prognosis of cancer patients. J Balkan Union Oncol.

[CR2] Andersen BLYHC, Farrar WB, Golden-Kreutz DM, Emery CF, Thornton LM (2008). Psychologic intervention improves survival for breast cancer patients: a randomized clinical trial. Cancer.

[CR3] Antoni MHLSK, Cole SW, Dhabhar FS, Sephton SE, McDonald PG, Stefanek M, Sood AK (2006). The influence of bio-behavioural factors on tumour biology: pathways and mechanisms. Nat Rev.

[CR4] Åsberg MNA, Leopardi R, Rylander G, Peterson U, Wilczek L, Källmén H, Ekstedt M, Åkerstedt T, Lekander M, Ekman R (2009). Novel biochemical markers of psychosocial stress in women. PLoS One.

[CR5] Barrera MA, Andrews GS, Saunders F (2008). Factors related to changes in cognitive, educational and visual motor integration in children who undergo hematopoietic stem cell transplant. J Pediatr Psychol.

[CR6] Best MSR, Catania L, Kazak AE (2001). Parental distress during pediatric leukemia and posttraumatic stress symptoms (PTSS) after treatment ends. J Pediatr Psychol.

[CR7] Colon-Echevarria CBL-CR, Aquino-Acevedo AN, Armaiz-Pena GN (2019). Neuroendocrine regulation of tumor-associated immune cells. Front Oncol.

[CR8] Costanzo ESSAK, Lutgendorf SK (2011). Biobehavioral influences on cancer progression. Immunol Allergy Clin North Am.

[CR9] Dai SMY, Wang Y, Xiang B, Liao Q, Zhou M (2020). Chronic stress promotes cancer development. Front Oncol.

[CR10] Feichtl RERB, Tallamy B, Cairo MS, Sands SA (2010). Concordance of quality of life assessments following pediatric hematopoietic stem cell transplantation. Psychooncology.

[CR11] Felder-Puig RDGA, Waldenmair M, Norden P, Winter A, Gadner H, Topf R (2006). Health-related quality of life of pediatric patients receiving allogeneic stem cell or bone marrow transplantation: results of a longitudinal, multi-center study. Bone Marrow Transplant.

[CR12] Gianinazzi ME, Rueegg CS, Wengenroth L, Bergstraesser E, Rischewski J, Ammann RA, Kuehni CE, Michel G (2013). Adolescent survivors of childhood cancer: are they vulnerable for psychological distress?. Psychooncology.

[CR13] Giese-Davis JCK, Rancourt KM, Neri E, Kraemer HC, Spiegel D (2011). Decrease in depression symptoms is associated with longer survival in patients with metastatic breast cancer: a secondary analysis. J Clin Oncol.

[CR14] Gratwohl A, Baldomero H, Aljurf M, Pasquini MC, Bouzas LF, Yoshimi A, Szer J, Lipton J, Schwendener A, Gratwohl M, Frauendorfer K, Niederwieser D, Horowitz M, Kodera Y, for the Worldwide Network of Blood and Marrow Transplantation (2010). Hematopoietic stem cell transplantation. A global perspective. J Am Med Assoc.

[CR15] Jacobsen PB, Donovan KA, Trask PC, Fleishman SB, Zabora J, Baker F, Holland JC (2005). Screening for psychologic distress in ambulatory cancer patients. Cancer.

[CR16] Kazak AE, Abrams AN, Banks J, Christofferson J, DiDonato S, Grootenhuis MA, Kabour M, Madan-Swain A, Patel SK, Zadeh S, Kupst MJ (2015). Psychosocial assessment as a standard of care in pediatric cancer. Pediatr Blood Cancer.

[CR17] Kuchler TBB, Rappat S, Henne-Bruns D, Wood-Dauphinee S (2007). Impact of psychotherapeutic support for patients with gastrointestinal cancer undergoing surgery: 10-year survival results of a randomized trial. J Clin Oncol.

[CR18] Lauer AL (2015). Treatment of anxiety and depression in adolescents and young adults with cancer. J Pediatr Oncol Nurs.

[CR19] Lazor T, Tigelaar L, Pole JD, De Souza C, Tomlinson D, Sung L (2017). Instruments to measure anxiety in children, adolescents, and young adults with cancer: a systematic review. Support Care Cancer.

[CR20] Lazor T, De Souza C, Urquhart R, Serhal E, R GA,  (2020). Few guidelines offer recommendations on how to assess and manage anxiety and distress in children with cancer: a content analysis. Support Care Cancer.

[CR21] Levine DRNKV, Talleur AC, Snyder A, Kaye EC, Baker JN (2020). Thoughts from the threshold: patient and family hopes, Fears, values, and goals at the onset of pediatric hematopoietic cell transplantation. Bone Marrow Transplant.

[CR22] McGregor BA, Syrjala KL, Dolan ED, Langer SL, Redman M (2013). The effect of pre-transplant distress on immune reconstitution among adult autologous hematopoietic cell transplantation patients. Brain Behav Immunity.

[CR23] Mehnert AMD, Lehmann C, Koch U (2006). Die deutsche VersiondesNCCN Distress-Thermometers Empirische Prüfung eines Screening-Instruments zur Erfassung psychosozialer Belastung bei Krebspatienten Zeitschrift für Psychiatrie. Psychologie Und Psychotherapie.

[CR24] Meyers CA, Weitzner M, Byrne K, Valentine A, Champlin RE, Przepiorka D (1994). Evaluation of the neurobehavioral functioning of patients before, during, and after bone marrow transplantation. J Clin Oncol.

[CR25] Myers RM, Balsamo L, Lu X, Devidas M, Hunger SP, Carroll WL, Winick NJ, Maloney KW, Kadan-Lottick NS (2014). A prospective study of anxiety, depression, and behavioral changes in the first year after a diagnosis of childhood acute lymphoblastic leukemia. Cancer.

[CR26] Nadolnik LI (2011). Stress and the thyroid gland. Biochem (moscow) Suppl Ser B Biomed Chem.

[CR27] Nelson AEMMS, Belyea MJ (1997). Coping and support effects on mothers' stress responses to their child's hematopoietic stem cell transplantation. J Pediatr Oncol Nurs.

[CR28] Patel SK, Mullins W, Turk A, Dekel N, Kinjo C, Sato JK (2010). Distress screening, rater agreement, and services in pediatric oncology. Psychooncology.

[CR29] Patel SK, Mullins W, Turk A, Dekel N, Kinjo C, Sato JK (2011). Distress screening, rater agreement, and services in pediatric oncology. Psychooncology.

[CR30] Patel SK, Fernandez N, Wong AL, Mullins W, Turk A, Dekel N, Smith M, Ferrell B (2014). Changes in self-reported distress in end-of-life pediatric cancer patients and their parents using the pediatric distress thermometer. Psychooncology.

[CR31] Patel SK, Kim SH, Johansen C, Mullins W, Nolty A, Fernandez N, Delgado N, Folbrecht J, Dekel N, Meier A (2021). Threshold score for the self-report Pediatric Distress Thermometer Rating Scale in childhood cancer patients. Psychooncology.

[CR32] Riva R, Forinder U, Arvidson J, Mellgren K, Toporski J, Winiarski J, Norberg AL (2014). Patterns of psychological responses in parents of children that underwent stem cell transplantation. Psychooncology.

[CR33] Schulte F, Russell KB, Pelletier W, Scott-Lane L, Guilcher GMT, Strother D, Dewey D (2019). Screening for psychosocial distress in pediatric cancer patients: an examination of feasibility in a single institution. Pediatr Hematol Oncol.

[CR34] Seelisch J, Sung L, Kelly MJ, Raybin JL, Beauchemin M, Dvorak CC, Patterson KK, Nieder ML, Noll RB, Thackray J, Ullrich NJ, Cabral S, Dupuis LL, Robinson PD (2018). Identifying clinical practice guidelines for the supportive care of children with cancer: a report from the Children'sOncology Group. Pediatr Blood Cancer.

[CR35] Vissoci Reiche EM, Morimoto HK, Nunes SM (2005). Stress and depression-induced immune dysfunction: implications for the development and progression of cancer. Int Rev Psychiatry.

[CR36] Wiener L, Battles H, Zadeh S, Widemann BC, Pao M (2017). Validity, specificity, feasibility and acceptability of a brief pediatric distress thermometer in outpatient clinics. Psychooncology.

